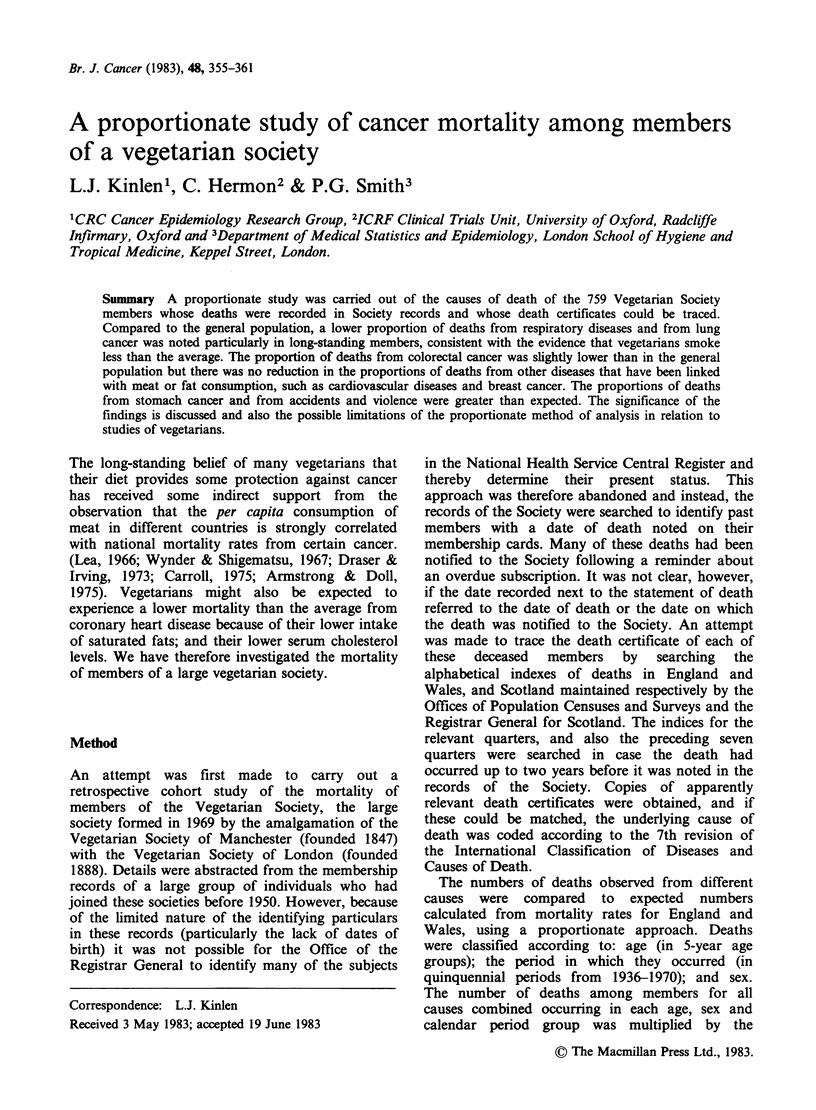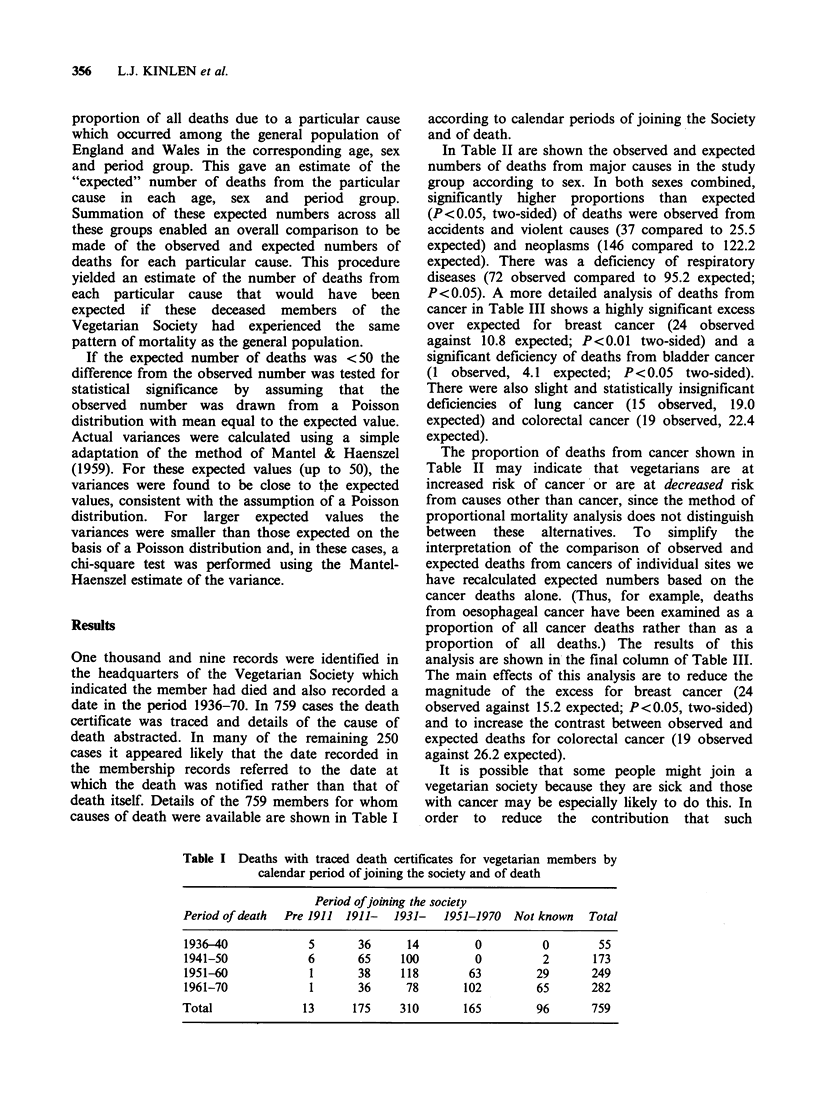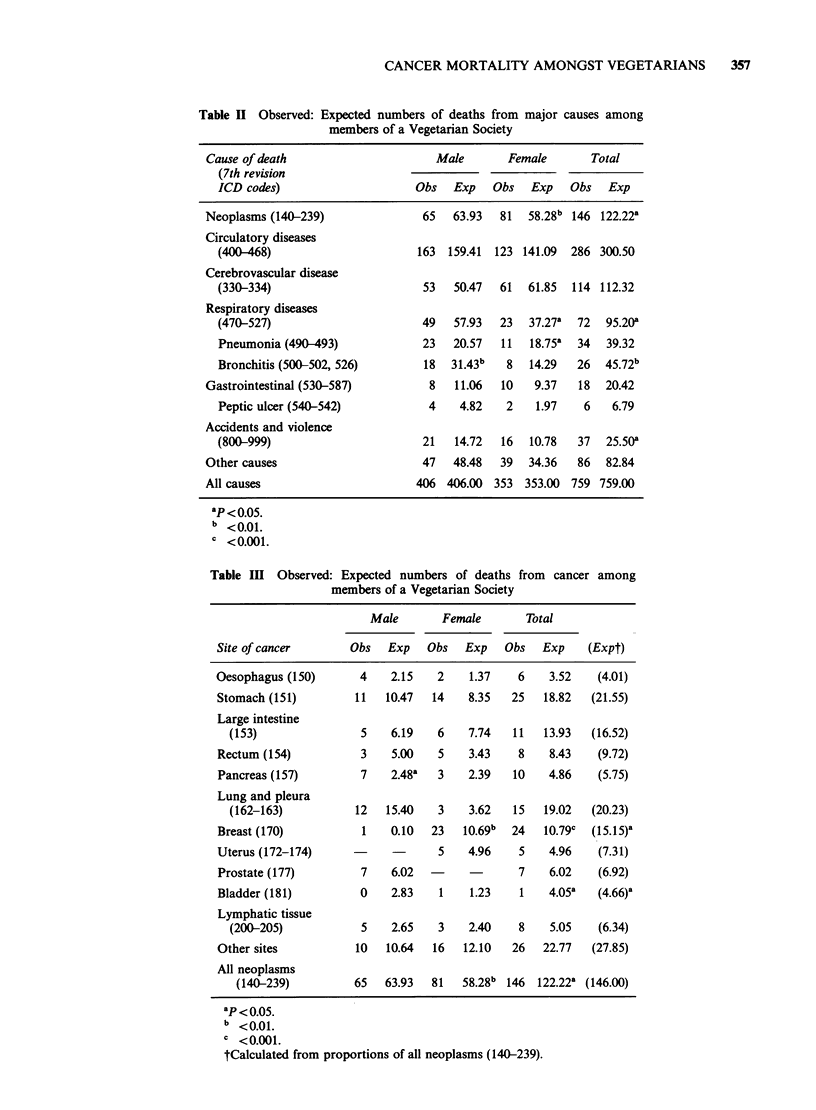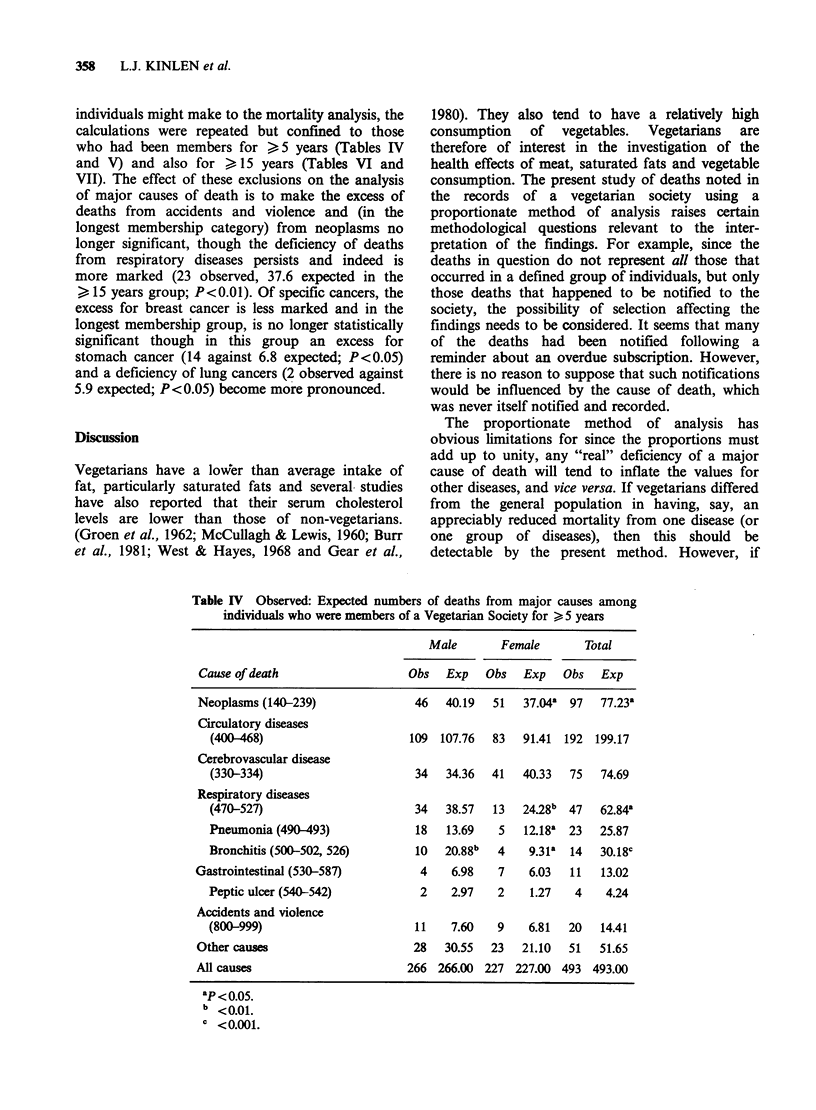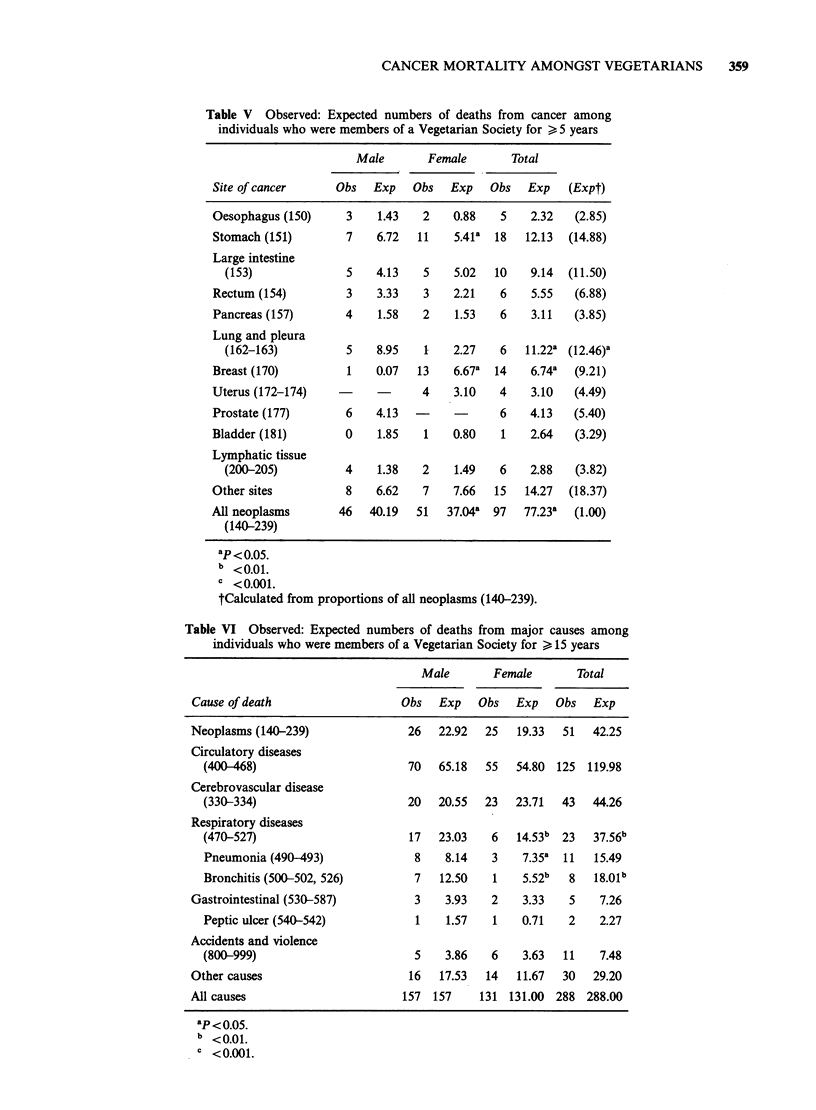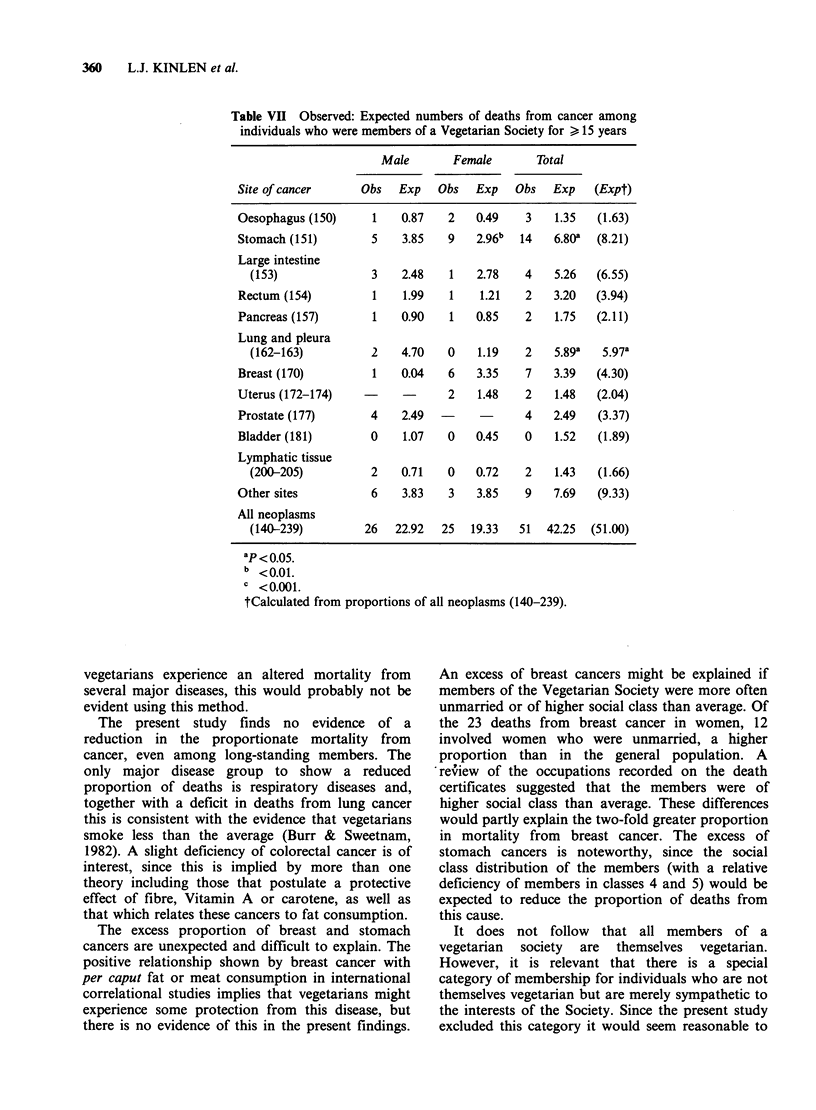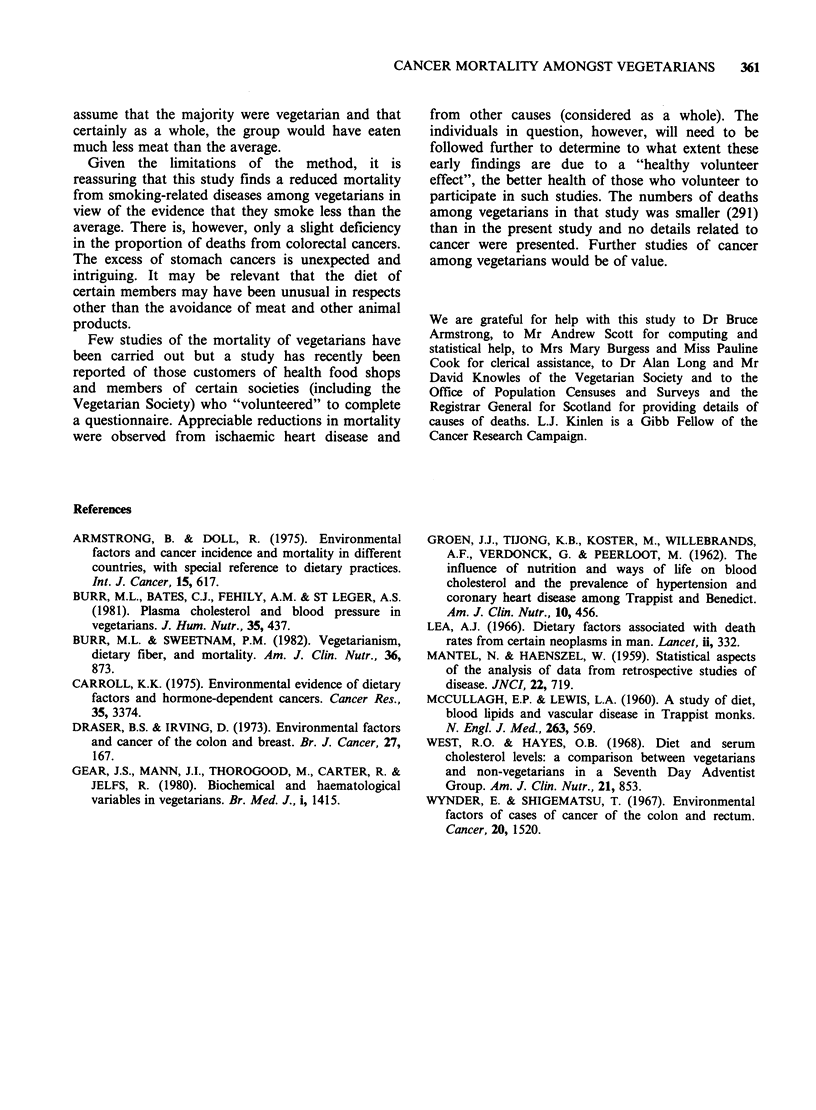# A proportionate study of cancer mortality among members of a vegetarian society.

**DOI:** 10.1038/bjc.1983.200

**Published:** 1983-09

**Authors:** L. J. Kinlen, C. Hermon, P. G. Smith

## Abstract

A proportionate study was carried out of the causes of death of the 759 Vegetarian Society members whose deaths were recorded in Society records and whose death certificates could be traced. Compared to the general population, a lower proportion of deaths from respiratory diseases and from lung cancer was noted particularly in long-standing members, consistent with the evidence that vegetarians smoke less than the average. The proportion of deaths from colorectal cancer was slightly lower than in the general population but there was no reduction in the proportions of deaths from other diseases that have been linked with meat or fat consumption, such as cardiovascular diseases and breast cancer. The proportions of deaths from stomach cancer and from accidents and violence were greater than expected. The significance of the findings is discussed and also the possible limitations of the proportionate method of analysis in relation to studies of vegetarians.


*Br. J. Cancer* (1983), **48**, 355–361

### A proportionate study of cancer mortality among members of a vegetarian society

L.J. Kinlen[1], C. Hermon[2] & P.G. Smith[3]

[1]*CRC Cancer Epidemiology Research Group,* [2]*ICRF Clinical Trials Unit, University of Oxford, Radcliffe Infirmary, Oxford and* [3]*Department of Medical Statistics and Epidemiology, London School of Hygiene and Tropical Medicine, Keppel Street, London.*

**Summary** A proportionate study was carried out of the causes of death of the 759 Vegetarian Society members whose deaths were recorded in Society records and whose death certificates could be traced. Compared to the general population, a lower proportion of deaths from respiratory diseases and from lung cancer was noted particularly in long-standing members, consistent with the evidence that vegetarians smoke less than the average. The proportion of deaths from colorectal cancer was slightly lower than in the general population but there was no reduction in the proportions of deaths from other diseases that have been linked with meat or fat consumption, such as cardiovascular diseases and breast cancer. The proportions of deaths from stomach cancer and from accidents and violence were greater than expected. The significance of the findings is discussed and also the possible limitations of the proportionate method of analysis in relation to studies of vegetarians.

The long-standing belief of many vegetarians that their diet provides some protection against cancer has received some indirect support from the observation that the *per capita* consumption of meat in different countries is strongly correlated with national mortality rates from certain cancer. (Lea, 1966; Wynder & Shigematsu, 1967; Draser & Irving, 1973; Carroll, 1975; Armstrong & Doll, 1975). Vegetarians might also be expected to experience a lower mortality than the average from coronary heart disease because of their lower intake of saturated fats; and their lower serum cholesterol levels. We have therefore investigated the mortality of members of a large vegetarian society.

### Method

An attempt was first made to carry out a retrospective cohort study of the mortality of members of the Vegetarian Society, the large society formed in 1969 by the amalgamation of the Vegetarian Society of Manchester (founded 1847) with the Vegetarian Society of London (founded 1888). Details were abstracted from the membership records of a large group of individuals who had joined these societies before 1950. However, because of the limited nature of the identifying particulars in these records (particularly the lack of dates of birth) it was not possible for the Office of the Registrar General to identify many of the subjects in the National Health Service Central Register and thereby determine their present status. This approach was therefore abandoned and instead, the records of the Society were searched to identify past members with a date of death noted on their membership cards. Many of these deaths had been notified to the Society following a reminder about an overdue subscription. It was not clear, however, if the date recorded next to the statement of death referred to the date of death or the date on which the death was notified to the Society. An attempt was made to trace the death certificate of each of these deceased members by searching the alphabetical indexes of deaths in England and Wales, and Scotland maintained respectively by the Offices of Population Censuses and Surveys and the Registrar General for Scotland. The indices for the relevant quarters, and also the preceding seven quarters were searched in case the death had occurred up to two years before it was noted in the records of the Society. Copies of apparently relevant death certificates were obtained, and if these could be matched, the underlying cause of death was coded according to the 7th revision of the International Classification of Diseases and Causes of Death.

The numbers of deaths observed from different causes were compared to expected numbers calculated from mortality rates for England and Wales, using a proportionate approach. Deaths were classified according to: age (in 5-year age groups); the period in which they occurred (in quinquennial periods from 1936–1970); and sex. The number of deaths among members for all causes combined occurring in each age, sex and calendar period group was multiplied by the

Correspondence: L.J. Kinlen

Received 3 May 1983; accepted 19 June 1983

© The Macmillan Press Ltd., 1983.

proportion of all deaths due to a particular cause which occurred among the general population of England and Wales in the corresponding age, sex and period group. This gave an estimate of the "expected" number of deaths from the particular cause in each age, sex and period group. Summation of these expected numbers across all these groups enabled an overall comparison to be made of the observed and expected numbers of deaths for each particular cause. This procedure yielded an estimate of the number of deaths from each particular cause that would have been expected if these deceased members of the Vegetarian Society had experienced the same pattern of mortality as the general population.

If the expected number of deaths was $<50$ the difference from the observed number was tested for statistical significance by assuming that the observed number was drawn from a Poisson distribution with mean equal to the expected value. Actual variances were calculated using a simple adaptation of the method of Mantel & Haenszel (1959). For these expected values (up to 50), the variances were found to be close to the expected values, consistent with the assumption of a Poisson distribution. For larger expected values the variances were smaller than those expected on the basis of a Poisson distribution and, in these cases, a chi-square test was performed using the Mantel-Haenszel estimate of the variance.

### Results

One thousand and nine records were identified in the headquarters of the Vegetarian Society which indicated the member had died and also recorded a date in the period 1936–70. In 759 cases the death certificate was traced and details of the cause of death abstracted. In many of the remaining 250 cases it appeared likely that the date recorded in the membership records referred to the date at which the death was notified rather than that of death itself. Details of the 759 members for whom causes of death were available are shown in Table I according to calendar periods of joining the Society and of death.

In Table II are shown the observed and expected numbers of deaths from major causes in the study group according to sex. In both sexes combined, significantly higher proportions than expected ($P<0.05$, two-sided) of deaths were observed from accidents and violent causes (37 compared to 25.5 expected) and neoplasms (146 compared to 122.2 expected). There was a deficiency of respiratory diseases (72 observed compared to 95.2 expected; $P<0.05$). A more detailed analysis of deaths from cancer in Table III shows a highly significant excess over expected for breast cancer (24 observed against 10.8 expected; $P<0.01$ two-sided) and a significant deficiency of deaths from bladder cancer (1 observed, 4.1 expected; $P<0.05$ two-sided). There were also slight and statistically insignificant deficiencies of lung cancer (15 observed, 19.0 expected) and colorectal cancer (19 observed, 22.4 expected).

The proportion of deaths from cancer shown in Table II may indicate that vegetarians are at increased risk of cancer or are at *decreased* risk from causes other than cancer, since the method of proportional mortality analysis does not distinguish between these alternatives. To simplify the interpretation of the comparison of observed and expected deaths from cancers of individual sites we have recalculated expected numbers based on the cancer deaths alone. (Thus, for example, deaths from oesophageal cancer have been examined as a proportion of all cancer deaths rather than as a proportion of all deaths.) The results of this analysis are shown in the final column of Table III. The main effects of this analysis are to reduce the magnitude of the excess for breast cancer (24 observed against 15.2 expected; $P<0.05$, two-sided) and to increase the contrast between observed and expected deaths for colorectal cancer (19 observed against 26.2 expected).

It is possible that some people might join a vegetarian society because they are sick and those with cancer may be especially likely to do this. In order to reduce the contribution that such

**Table I** Deaths with traced death certificates for vegetarian members by calendar period of joining the society and of death

| | *Period of joining the society* | | | | | |
|---|---|---|---|---|---|---|
| *Period of death* | *Pre 1911* | *1911–* | *1931–* | *1951–1970* | *Not known* | *Total* |
| 1936–40 | 5 | 36 | 14 | 0 | 0 | 55 |
| 1941–50 | 6 | 65 | 100 | 0 | 2 | 173 |
| 1951–60 | 1 | 38 | 118 | 63 | 29 | 249 |
| 1961–70 | 1 | 36 | 78 | 102 | 65 | 282 |
| Total | 13 | 175 | 310 | 165 | 96 | 759 |

**Table II** Observed: Expected numbers of deaths from major causes among members of a Vegetarian Society

| *Cause of death (7th revision ICD codes)* | *Male* | | *Female* | | *Total* | |
|---|---|---|---|---|---|---|
| | *Obs* | *Exp* | *Obs* | *Exp* | *Obs* | *Exp* |
| Neoplasms (140–239) | 65 | 63.93 | 81 | $58.28^{b}$ | 146 | $122.22^{a}$ |
| Circulatory diseases (400–468) | 163 | 159.41 | 123 | 141.09 | 286 | 300.50 |
| Cerebrovascular disease (330–334) | 53 | 50.47 | 61 | 61.85 | 114 | 112.32 |
| Respiratory diseases (470–527) | 49 | 57.93 | 23 | $37.27^{a}$ | 72 | $95.20^{a}$ |
| Pneumonia (490–493) | 23 | 20.57 | 11 | $18.75^{a}$ | 34 | 39.32 |
| Bronchitis (500–502, 526) | 18 | $31.43^{b}$ | 8 | 14.29 | 26 | $45.72^{b}$ |
| Gastrointestinal (530–587) | 8 | 11.06 | 10 | 9.37 | 18 | 20.42 |
| Peptic ulcer (540–542) | 4 | 4.82 | 2 | 1.97 | 6 | 6.79 |
| Accidents and violence (800–999) | 21 | 14.72 | 16 | 10.78 | 37 | $25.50^{a}$ |
| Other causes | 47 | 48.48 | 39 | 34.36 | 86 | 82.84 |
| All causes | 406 | 406.00 | 353 | 353.00 | 759 | 759.00 |

$^{a}P<0.05$.
$^{b}$ $<0.01$.
$^{c}$ $<0.001$.

**Table III** Observed: Expected numbers of deaths from cancer among members of a Vegetarian Society

| | *Male* | | *Female* | | *Total* | | |
|---|---|---|---|---|---|---|---|
| *Site of cancer* | *Obs* | *Exp* | *Obs* | *Exp* | *Obs* | *Exp* | *(Exp†)* |
| Oesophagus (150) | 4 | 2.15 | 2 | 1.37 | 6 | 3.52 | (4.01) |
| Stomach (151) | 11 | 10.47 | 14 | 8.35 | 25 | 18.82 | (21.55) |
| Large intestine (153) | 5 | 6.19 | 6 | 7.74 | 11 | 13.93 | (16.52) |
| Rectum (154) | 3 | 5.00 | 5 | 3.43 | 8 | 8.43 | (9.72) |
| Pancreas (157) | 7 | $2.48^{a}$ | 3 | 2.39 | 10 | 4.86 | (5.75) |
| Lung and pleura (162–163) | 12 | 15.40 | 3 | 3.62 | 15 | 19.02 | (20.23) |
| Breast (170) | 1 | 0.10 | 23 | $10.69^{b}$ | 24 | $10.79^{c}$ | $(15.15)^{a}$ |
| Uterus (172–174) | — | — | 5 | 4.96 | 5 | 4.96 | (7.31) |
| Prostate (177) | 7 | 6.02 | — | — | 7 | 6.02 | (6.92) |
| Bladder (181) | 0 | 2.83 | 1 | 1.23 | 1 | $4.05^{a}$ | $(4.66)^{a}$ |
| Lymphatic tissue (200–205) | 5 | 2.65 | 3 | 2.40 | 8 | 5.05 | (6.34) |
| Other sites | 10 | 10.64 | 16 | 12.10 | 26 | 22.77 | (27.85) |
| All neoplasms (140–239) | 65 | 63.93 | 81 | $58.28^{b}$ | 146 | $122.22^{a}$ | (146.00) |

$^{a}P<0.05$.
$^{b}$ $<0.01$.
$^{c}$ $<0.001$.
†Calculated from proportions of all neoplasms (140–239).

individuals might make to the mortality analysis, the calculations were repeated but confined to those who had been members for ⩾5 years (Tables IV and V) and also for ⩾15 years (Tables VI and VII). The effect of these exclusions on the analysis of major causes of death is to make the excess of deaths from accidents and violence and (in the longest membership category) from neoplasms no longer significant, though the deficiency of deaths from respiratory diseases persists and indeed is more marked (23 observed, 37.6 expected in the ⩾15 years group; $P<0.01$). Of specific cancers, the excess for breast cancer is less marked and in the longest membership group, is no longer statistically significant though in this group an excess for stomach cancer (14 against 6.8 expected; $P<0.05$) and a deficiency of lung cancers (2 observed against 5.9 expected; $P<0.05$) become more pronounced.

### Discussion

Vegetarians have a lower than average intake of fat, particularly saturated fats and several studies have also reported that their serum cholesterol levels are lower than those of non-vegetarians. (Groen *et al.*, 1962; McCullagh & Lewis, 1960; Burr *et al.*, 1981; West & Hayes, 1968 and Gear *et al.*, 1980). They also tend to have a relatively high consumption of vegetables. Vegetarians are therefore of interest in the investigation of the health effects of meat, saturated fats and vegetable consumption. The present study of deaths noted in the records of a vegetarian society using a proportionate method of analysis raises certain methodological questions relevant to the interpretation of the findings. For example, since the deaths in question do not represent *all* those that occurred in a defined group of individuals, but only those deaths that happened to be notified to the society, the possibility of selection affecting the findings needs to be considered. It seems that many of the deaths had been notified following a reminder about an overdue subscription. However, there is no reason to suppose that such notifications would be influenced by the cause of death, which was never itself notified and recorded.

The proportionate method of analysis has obvious limitations for since the proportions must add up to unity, any "real" deficiency of a major cause of death will tend to inflate the values for other diseases, and *vice versa*. If vegetarians differed from the general population in having, say, an appreciably reduced mortality from one disease (or one group of diseases), then this should be detectable by the present method. However, if

**Table IV** Observed: Expected numbers of deaths from major causes among individuals who were members of a Vegetarian Society for ⩾5 years

| | *Male* | | *Female* | | *Total* | |
|---|---|---|---|---|---|---|
| *Cause of death* | *Obs* | *Exp* | *Obs* | *Exp* | *Obs* | *Exp* |
| Neoplasms (140–239) | 46 | 40.19 | 51 | 37.04[a] | 97 | 77.23[a] |
| Circulatory diseases (400–468) | 109 | 107.76 | 83 | 91.41 | 192 | 199.17 |
| Cerebrovascular disease (330–334) | 34 | 34.36 | 41 | 40.33 | 75 | 74.69 |
| Respiratory diseases (470–527) | 34 | 38.57 | 13 | 24.28[b] | 47 | 62.84[a] |
| Pneumonia (490–493) | 18 | 13.69 | 5 | 12.18[a] | 23 | 25.87 |
| Bronchitis (500–502, 526) | 10 | 20.88[b] | 4 | 9.31[a] | 14 | 30.18[c] |
| Gastrointestinal (530–587) | 4 | 6.98 | 7 | 6.03 | 11 | 13.02 |
| Peptic ulcer (540–542) | 2 | 2.97 | 2 | 1.27 | 4 | 4.24 |
| Accidents and violence (800–999) | 11 | 7.60 | 9 | 6.81 | 20 | 14.41 |
| Other causes | 28 | 30.55 | 23 | 21.10 | 51 | 51.65 |
| All causes | 266 | 266.00 | 227 | 227.00 | 493 | 493.00 |

[a] $P<0.05$.
[b] $<0.01$.
[c] $<0.001$.

**Table V** Observed: Expected numbers of deaths from cancer among individuals who were members of a Vegetarian Society for ⩾5 years

| | *Male* | | *Female* | | *Total* | | |
|---|---|---|---|---|---|---|---|
| *Site of cancer* | *Obs* | *Exp* | *Obs* | *Exp* | *Obs* | *Exp* | *(Exp†)* |
| Oesophagus (150) | 3 | 1.43 | 2 | 0.88 | 5 | 2.32 | (2.85) |
| Stomach (151) | 7 | 6.72 | 11 | 5.41[a] | 18 | 12.13 | (14.88) |
| Large intestine (153) | 5 | 4.13 | 5 | 5.02 | 10 | 9.14 | (11.50) |
| Rectum (154) | 3 | 3.33 | 3 | 2.21 | 6 | 5.55 | (6.88) |
| Pancreas (157) | 4 | 1.58 | 2 | 1.53 | 6 | 3.11 | (3.85) |
| Lung and pleura (162–163) | 5 | 8.95 | 1 | 2.27 | 6 | 11.22[a] | (12.46)[a] |
| Breast (170) | 1 | 0.07 | 13 | 6.67[a] | 14 | 6.74[a] | (9.21) |
| Uterus (172–174) | — | — | 4 | 3.10 | 4 | 3.10 | (4.49) |
| Prostate (177) | 6 | 4.13 | — | — | 6 | 4.13 | (5.40) |
| Bladder (181) | 0 | 1.85 | 1 | 0.80 | 1 | 2.64 | (3.29) |
| Lymphatic tissue (200–205) | 4 | 1.38 | 2 | 1.49 | 6 | 2.88 | (3.82) |
| Other sites | 8 | 6.62 | 7 | 7.66 | 15 | 14.27 | (18.37) |
| All neoplasms (140–239) | 46 | 40.19 | 51 | 37.04[a] | 97 | 77.23[a] | (1.00) |

[a] $P<0.05$.
[b] $<0.01$.
[c] $<0.001$.
†Calculated from proportions of all neoplasms (140–239).

**Table VI** Observed: Expected numbers of deaths from major causes among individuals who were members of a Vegetarian Society for ⩾15 years

| | *Male* | | *Female* | | *Total* | |
|---|---|---|---|---|---|---|
| *Cause of death* | *Obs* | *Exp* | *Obs* | *Exp* | *Obs* | *Exp* |
| Neoplasms (140–239) | 26 | 22.92 | 25 | 19.33 | 51 | 42.25 |
| Circulatory diseases (400–468) | 70 | 65.18 | 55 | 54.80 | 125 | 119.98 |
| Cerebrovascular disease (330–334) | 20 | 20.55 | 23 | 23.71 | 43 | 44.26 |
| Respiratory diseases (470–527) | 17 | 23.03 | 6 | 14.53[b] | 23 | 37.56[b] |
| Pneumonia (490–493) | 8 | 8.14 | 3 | 7.35[a] | 11 | 15.49 |
| Bronchitis (500–502, 526) | 7 | 12.50 | 1 | 5.52[b] | 8 | 18.01[b] |
| Gastrointestinal (530–587) | 3 | 3.93 | 2 | 3.33 | 5 | 7.26 |
| Peptic ulcer (540–542) | 1 | 1.57 | 1 | 0.71 | 2 | 2.27 |
| Accidents and violence (800–999) | 5 | 3.86 | 6 | 3.63 | 11 | 7.48 |
| Other causes | 16 | 17.53 | 14 | 11.67 | 30 | 29.20 |
| All causes | 157 | 157 | 131 | 131.00 | 288 | 288.00 |

[a] $P<0.05$.
[b] $<0.01$.
[c] $<0.001$.

**Table VII** Observed: Expected numbers of deaths from cancer among individuals who were members of a Vegetarian Society for ⩾15 years

| | *Male* | | *Female* | | *Total* | | |
|---|---|---|---|---|---|---|---|
| *Site of cancer* | *Obs* | *Exp* | *Obs* | *Exp* | *Obs* | *Exp* | *(Exp†)* |
| Oesophagus (150) | 1 | 0.87 | 2 | 0.49 | 3 | 1.35 | (1.63) |
| Stomach (151) | 5 | 3.85 | 9 | 2.96[b] | 14 | 6.80[a] | (8.21) |
| Large intestine (153) | 3 | 2.48 | 1 | 2.78 | 4 | 5.26 | (6.55) |
| Rectum (154) | 1 | 1.99 | 1 | 1.21 | 2 | 3.20 | (3.94) |
| Pancreas (157) | 1 | 0.90 | 1 | 0.85 | 2 | 1.75 | (2.11) |
| Lung and pleura (162–163) | 2 | 4.70 | 0 | 1.19 | 2 | 5.89[a] | 5.97[a] |
| Breast (170) | 1 | 0.04 | 6 | 3.35 | 7 | 3.39 | (4.30) |
| Uterus (172–174) | — | — | 2 | 1.48 | 2 | 1.48 | (2.04) |
| Prostate (177) | 4 | 2.49 | — | — | 4 | 2.49 | (3.37) |
| Bladder (181) | 0 | 1.07 | 0 | 0.45 | 0 | 1.52 | (1.89) |
| Lymphatic tissue (200–205) | 2 | 0.71 | 0 | 0.72 | 2 | 1.43 | (1.66) |
| Other sites | 6 | 3.83 | 3 | 3.85 | 9 | 7.69 | (9.33) |
| All neoplasms (140–239) | 26 | 22.92 | 25 | 19.33 | 51 | 42.25 | (51.00) |

[a]$P<0.05$.
[b] $<0.01$.
[c] $<0.001$.
†Calculated from proportions of all neoplasms (140–239).

vegetarians experience an altered mortality from several major diseases, this would probably not be evident using this method.

The present study finds no evidence of a reduction in the proportionate mortality from cancer, even among long-standing members. The only major disease group to show a reduced proportion of deaths is respiratory diseases and, together with a deficit in deaths from lung cancer this is consistent with the evidence that vegetarians smoke less than the average (Burr & Sweetnam, 1982). A slight deficiency of colorectal cancer is of interest, since this is implied by more than one theory including those that postulate a protective effect of fibre, Vitamin A or carotene, as well as that which relates these cancers to fat consumption.

The excess proportion of breast and stomach cancers are unexpected and difficult to explain. The positive relationship shown by breast cancer with *per caput* fat or meat consumption in international correlational studies implies that vegetarians might experience some protection from this disease, but there is no evidence of this in the present findings. An excess of breast cancers might be explained if members of the Vegetarian Society were more often unmarried or of higher social class than average. Of the 23 deaths from breast cancer in women, 12 involved women who were unmarried, a higher proportion than in the general population. A review of the occupations recorded on the death certificates suggested that the members were of higher social class than average. These differences would partly explain the two-fold greater proportion in mortality from breast cancer. The excess of stomach cancers is noteworthy, since the social class distribution of the members (with a relative deficiency of members in classes 4 and 5) would be expected to reduce the proportion of deaths from this cause.

It does not follow that all members of a vegetarian society are themselves vegetarian. However, it is relevant that there is a special category of membership for individuals who are not themselves vegetarian but are merely sympathetic to the interests of the Society. Since the present study excluded this category it would seem reasonable to assume that the majority were vegetarian and that certainly as a whole, the group would have eaten much less meat than the average.

Given the limitations of the method, it is reassuring that this study finds a reduced mortality from smoking-related diseases among vegetarians in view of the evidence that they smoke less than the average. There is, however, only a slight deficiency in the proportion of deaths from colorectal cancers. The excess of stomach cancers is unexpected and intriguing. It may be relevant that the diet of certain members may have been unusual in respects other than the avoidance of meat and other animal products.

Few studies of the mortality of vegetarians have been carried out but a study has recently been reported of those customers of health food shops and members of certain societies (including the Vegetarian Society) who "volunteered" to complete a questionnaire. Appreciable reductions in mortality were observed from ischaemic heart disease and from other causes (considered as a whole). The individuals in question, however, will need to be followed further to determine to what extent these early findings are due to a "healthy volunteer effect", the better health of those who volunteer to participate in such studies. The numbers of deaths among vegetarians in that study was smaller (291) than in the present study and no details related to cancer were presented. Further studies of cancer among vegetarians would be of value.

We are grateful for help with this study to Dr Bruce Armstrong, to Mr Andrew Scott for computing and statistical help, to Mrs Mary Burgess and Miss Pauline Cook for clerical assistance, to Dr Alan Long and Mr David Knowles of the Vegetarian Society and to the Office of Population Censuses and Surveys and the Registrar General for Scotland for providing details of causes of deaths. L.J. Kinlen is a Gibb Fellow of the Cancer Research Campaign.

### References

ARMSTRONG, B. & DOLL, R. (1975). Environmental factors and cancer incidence and mortality in different countries, with special reference to dietary practices. *Int. J. Cancer*, **15**, 617.

BURR, M.L., BATES, C.J., FEHILY, A.M. & ST LEGER, A.S. (1981). Plasma cholesterol and blood pressure in vegetarians. *J. Hum. Nutr.*, **35**, 437.

BURR, M.L. & SWEETNAM, P.M. (1982). Vegetarianism, dietary fiber, and mortality. *Am. J. Clin. Nutr.*, **36**, 873.

CARROLL, K.K. (1975). Environmental evidence of dietary factors and hormone-dependent cancers. *Cancer Res.*, **35**, 3374.

DRASER, B.S. & IRVING, D. (1973). Environmental factors and cancer of the colon and breast. *Br. J. Cancer*, **27**, 167.

GEAR, J.S., MANN, J.I., THOROGOOD, M., CARTER, R. & JELFS, R. (1980). Biochemical and haematological variables in vegetarians. *Br. Med. J.*, **i**, 1415.

GROEN, J.J., TIJONG, K.B., KOSTER, M., WILLEBRANDS, A.F., VERDONCK, G. & PEERLOOT, M. (1962). The influence of nutrition and ways of life on blood cholesterol and the prevalence of hypertension and coronary heart disease among Trappist and Benedict. *Am. J. Clin. Nutr.*, **10**, 456.

LEA, A.J. (1966). Dietary factors associated with death rates from certain neoplasms in man. *Lancet*, **ii**, 332.

MANTEL, N. & HAENSZEL, W. (1959). Statistical aspects of the analysis of data from retrospective studies of disease. *JNCI*, **22**, 719.

MCCULLAGH, E.P. & LEWIS, L.A. (1960). A study of diet, blood lipids and vascular disease in Trappist monks. *N. Engl. J. Med.*, **263**, 569.

WEST, R.O. & HAYES, O.B. (1968). Diet and serum cholesterol levels: a comparison between vegetarians and non-vegetarians in a Seventh Day Adventist Group. *Am. J. Clin. Nutr.*, **21**, 853.

WYNDER, E. & SHIGEMATSU, T. (1967). Environmental factors of cases of cancer of the colon and rectum. *Cancer*, **20**, 1520.